# Will human influences on evolutionary dynamics in the wild pervade the Anthropocene?

**DOI:** 10.1186/s12915-017-0476-1

**Published:** 2018-01-15

**Authors:** Fanie Pelletier, David W. Coltman

**Affiliations:** 1 Département de biologie, Université de Sherbrooke, Sherbrooke, QC J1K 2R1 Canada; 2 Department of Biological Sciences, University of Alberta, Edmonton, AB T6G 2E9 Canada

## Abstract

The five most pervasive anthropogenic threats to biodiversity are over-exploitation, habitat changes, climate change, invasive species, and pollution. Since all of these threats can affect intraspecific biodiversity—including genetic variation within populations—humans have the potential to induce contemporary microevolution in wild populations. We highlight recent empirical studies that have explored the effects of these anthropogenic threats to intraspecific biodiversity in the wild. We conclude that it is critical that we move towards a predictive framework that integrates a better understanding of contemporary microevolution to multiple threats to forecast the fate of natural populations in a changing world.

## Living in the Anthropocene

The human footprint on our planet is increasing at an unprecedented rate [1]. It has been suggested that we are now entering a new era, the Anthropocene, in which humans are one of the major drivers of environmental change [2]. Key anthropogenic challenges that wild populations face include increased habitat fragmentation and modification, over-exploitation for subsistence or sport hunting, and anthropogenic climate change [1, 3]. How wild populations will react to these new conditions is difficult to predict, although plastic responses, local adaptation, relocation and/or extinction are the likely local and global outcomes.

Organisms can respond most rapidly to environmental (natural or anthropogenic) change via plasticity or relocation. Phenotypic plasticity is the capacity of a genotype to produce different phenotypes in response to environmental changes [4]. For example, global climate change may affect how resources are distributed in time or space; in response organisms may alter their breeding timing to compensate for these changes [5, 6]. If plastic responses are not sufficient to cope with the new conditions, then organisms can also disperse to other habitats to follow historical conditions. This type of ecological response by populations and species has been widely documented in the context of climate changes. For example, a meta-analysis, including data on more than 1700 species, revealed significant range shifts averaging 6.1 km per decade towards the poles [7]. If populations face very drastic changes, however, they may be unable to compensate through ecological responses to the new conditions, suffer a decline in reproduction and survival, and ultimately go extinct unless they rapidly genetically adapt [8]. The process by which genetic adaptation occurs fast enough for species to cope with novel environmental conditions and prevent extinction is known as evolutionary rescue [9]. Laboratory experiments in microcosms suggest that in large populations of yeast, with minimal stochastic effects, evolutionary rescue takes approximately 25 generations to occur [10].

Humans are considered one of the major selective forces shaping species’ traits, often causing faster phenotypic change than many natural drivers [11–13], and human-driven trait changes have been observed all over the world [14]. Understanding how populations respond to human-induced selective pressures is therefore critical for both science and policy because our activities impact the ecology and evolution of wild species, and ultimately their persistence, compromising important ecosystem services [15, 16]. The goal of this review is threefold: first to outline how the five main recognized human threats to biodiversity present strong, synergistic drivers of microevolution in wild populations; second to discuss the burden of proof for detecting micro-evolutionary change at the phenotypic and/or genomic level and whether it has generally been met; and finally to provide some perspective on what is needed going forward.

## The big five: the nature of human-induced selective drivers

**Fig. 1.**
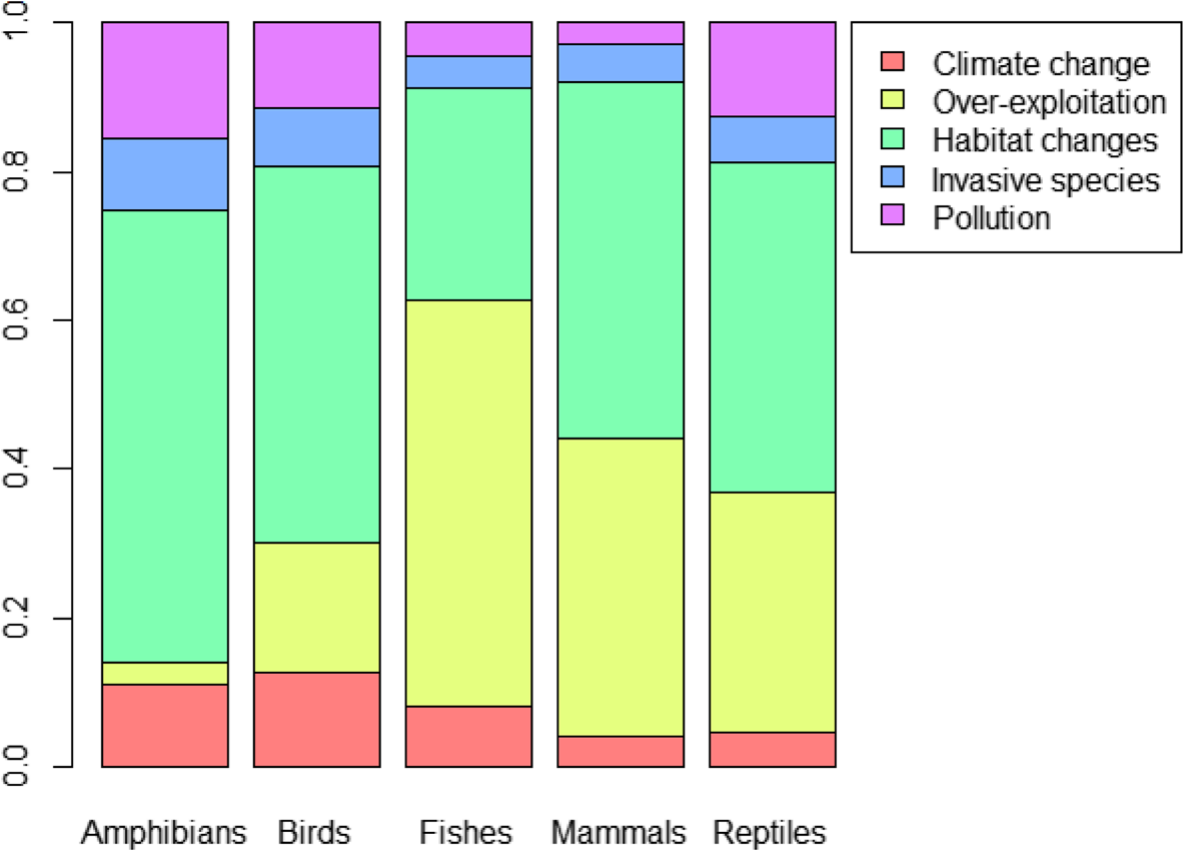
Main human threats to biodiversity. Relative importance of the five main human threats to biodiversity on 3564 wild populations according to taxonomic group (note that data on disease (N = 193) have been excluded). Figure modified from the Living Planet Report [1]

**Fig. 2.**
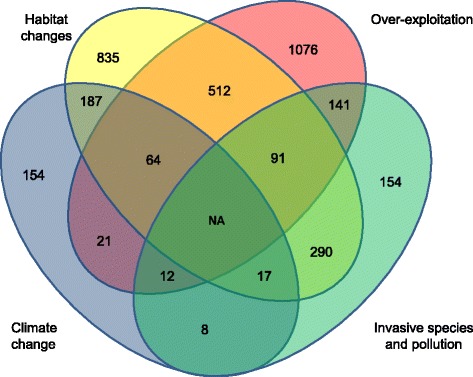
Number of populations affected by one or more human threats to biodiversity. Non-proportional Venn diagram illustrating the number of populations affected by one or more of the five main human threats to biodiversity. Note that data on invasive species and pollution have been combined to simplify the presentation, and that the Living Planet database registers a maximum of three threats per population. Data are from the Living Planet Report [1]. No data are available for the possible extent of the four influences combined (centre) as the database does not record more than three threats

**Fig. 3.**
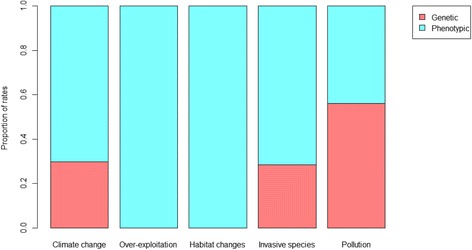
Published evidence for genetic trait changes in response to human activities. Proportion of study population traits that show rates of phenotypic change in response to the five main human threats to biodiversity for which there was evidence that the changes were at least partly genetic. Data are from version 4.04 of the trait database used in Alberti et al. [105]. We filtered the dataset to exclude pairwise allochronic contrasts and to include only cases were Haldane or Darwin rates were calculated. We also only retained rates measured between 1 and 300 generations, to illustrate only studies that have focused on microevolution to the five main threats discussed in this review (N_rates_ = 2239, N_studies_ = 124)

Human activities now impose the dominant pressures affecting the mortality and reproductive schedules of many species [3]. According to the Living Planet Report 2016 that compiles the human threats affecting over 3000 wild vertebrate populations, the main factors are over-exploitation, habitat degradation and loss, climate change, invasive species, and pollution [1]. Although the relative importance of each of these factors varies among taxa (Fig. 1), habitat changes (including loss and degradation) and over-exploitation account for 75% of the pressure on wild vertebrate populations [17]. More importantly, several of these threats affect wild populations simultaneously (Fig. 2) and their interactive effects likely accelerate their impacts on biodiversity [18]. For example, the rate of invasion by alien species is expected to increase with global climate change [19]. To illustrate the cumulative effects of human driven changes, we quantified the number of populations that face more than one threat in the Living Planet database [17]. Based on these data, 32% of the vertebrate populations surveyed experience at least two threats and 8% experience three of them (Fig. 2). The cumulative effect of these factors could either promote or limit the potential for evolutionary responses to occur. For example, endangered species are often reduced to small, isolated, and potentially genetically depauperate groups by anthropogenic habitat loss or habitat modifications, and are thus additionally sensitive to additive sources of mortality, such as invasive predators or disease [18, 20]. Although the Living Planet database only documents threats to vertebrate populations, phenotypic responses to all of these threats have been detected in various taxa (Fig. 3), suggesting that microevolutionary responses may be widespread. However, only a limited number of studies have access to genetic data to assess whether trait changes have a genetic basis (Fig. 3), but even when they do, it can be extremely difficult to distinguish genetic changes from plasticity [6, 21, 22]. Thus, understanding how human-driven selective pressures interact, and predicting their evolutionary impact, is a very challenging task. This raises the question of what evidence there is for microevolution in response to the main threats posed by human activity?

### Over-exploitation of wild populations

**Fig. 4.**
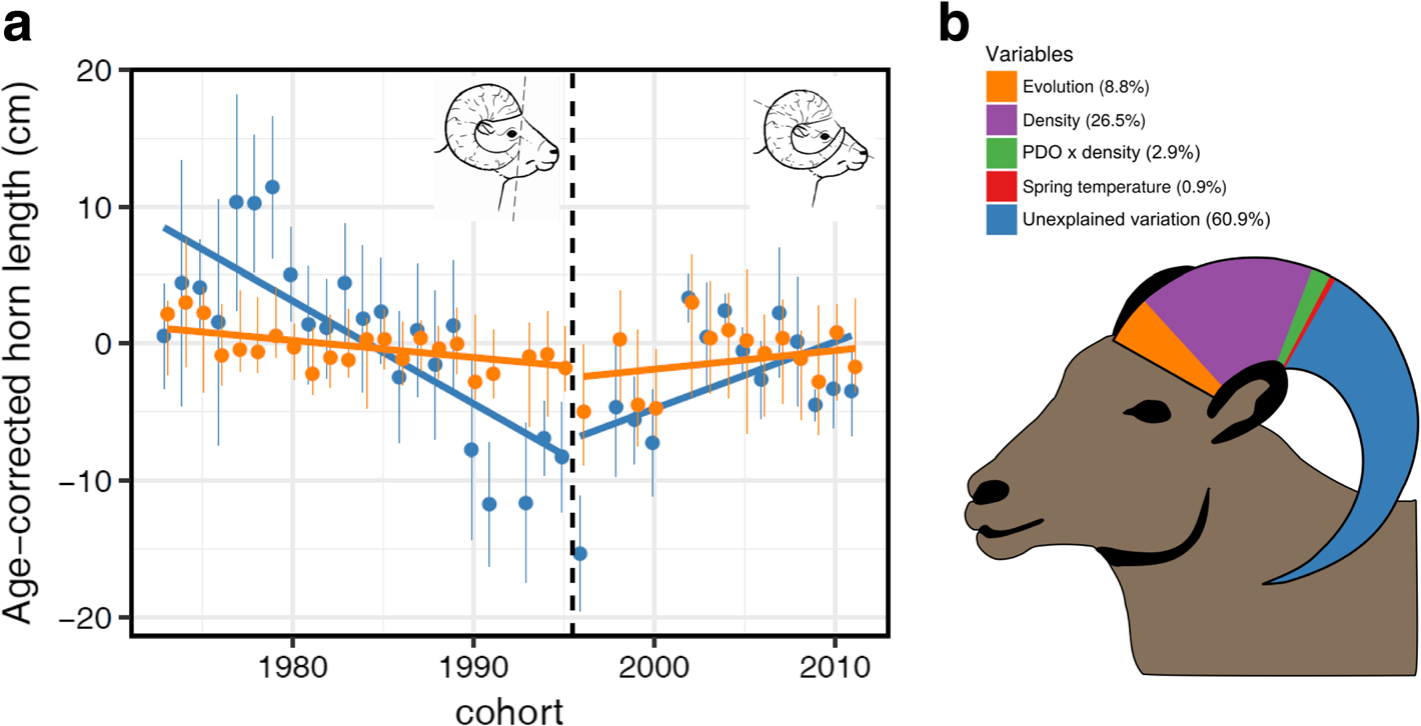
Evolutionary response to over-exploitation of horn size. A trophy hunted population of bighorn sheep at Ram Mountain, AB, Canada, individually monitored for more than four decades, experienced a decline in horn length, a trait with heritability of 0.36 [34]. **a** Quantitative genetic analyses revealed that part of this decline was genetically based [33, 34]. For the first 23 years of monitoring, rams could be legally harvested when their horn size achieved four-fifths of a full curl (see drawing *top left* in **a**). In 1996, the regulation was changed to a full curl (see drawing *top right* in **a**), allowing rams to survive longer into their peak reproductive years before reaching legal size. This change, along with the reduced availability of legal rams, decreased hunting pressure and the genetic decline ceased (panel **a** modified from [34]). Phenotypic (*blue dots* and *line*) and genetic (*orange dots* and *line*) estimates of age-corrected horn length of bighorn rams aged 2–4 years declined during the period of high hunting (1973–1995) and slowly recovered under low hunting pressure (1996–2011). This study conservatively suggests that microevolutionary change accounted for 2.62 cm of the observed decline in horn length during the period of high hunting pressure [34]. Douhard et al. [35] showed that approximately 8.8% of the variance in horn length at age 3 is explained by microevolutionary effects, estimated as the mean of the posterior distribution in breeding values for horn length over the entire study period (the variance decomposition is illustrated in proportion to the length of the sheep horn in **b**). Other major sources of variation illustrated in **b** include the Pacific decadal oscillation (*PDO*), a large scale climatic index, population density, and their interaction [35]

Over-exploitation is one of the main threats to biodiversity—humans often act as “super-predators” [23]. While the links between over-exploitation, abundance, extinction, and species-level biodiversity loss are perhaps obvious, several studies also reveal that humans are a powerful selective force affecting intraspecific biodiversity [12, 24]. For example, in a meta-analysis compiling data from 11,049 loci across 140 species of marine fishes, Pinksy and Palumbi [25] report a 12% reduction in allelic richness and 2% reduction in heterozygosity in overfished populations. Over-exploitation may cause genetic bottlenecks in historically abundant species, and thus directly impact intraspecific biodiversity. Harvesting may also impact the population structure and diversity of terrestrial wildlife [26, 27], and also induce hard selective pressure on morphological, behavioral, and life-history traits because of the non-random way in which humans remove certain phenotypes [13, 24, 28, 29]. In fact, phenotypic change seems to be faster when humans harvest wild populations compared to natural predators: a meta-analysis investigating the rates of phenotypic change in size-related and life-history traits in 40 harvested populations found that rates of change were 300% higher than in populations facing only natural selective factors [12]. Although this meta-analysis did not provide evidence that trait changes had a genetic basis, studies on size-selective fisheries [30–32] and trophy hunting [33, 34] suggest that changes are partially genetic. For example, two or three generations of intense trophy-hunting have led to a 2.6 cm genetic decline in horn length of bighorn rams (*Ovis canadensis*; Fig. 4a) and the breeding values for this trait accounted for 8.8% of variance in 3-year-old ram horn length (Fig. 4b). During that period, a legal ram, usually between 4 and 6 years old, faced a 40% probability of being shot during each subsequent hunting season [33], yet large horns in rams are not associated with reproductive success until after 7 years of age [35]. This genetic decline only stopped after a change in hunting regulations that reduced hunting intensity (Fig. 4a).

### Habitat changes

Loss of habitat, modifications in land use, degradation and fragmentation of habitat patches, and the increasing footprint of human infrastructure (hereafter referred to as “habitat change”) can all reduce population size and impede connectivity among populations. Habitat changes therefore impact effective population size, dispersal, and gene flow, which have significant implications for microevolutionary dynamics [36–38]. Reduced gene flow among small populations can lead to an increase in inbreeding, loss of diversity, and elevated extinction risk [39], but is also likely to reduce the potential for genetic rescue to occur [40]. Given that genetic variability is critical for adaptation to novel selective pressures, researchers have argued that reduced genetic diversity in small and isolated populations may limit their ability to adapt to novel selective pressures [20]. For example, a recent study on two meta-populations of the Glanville fritillary butterfly (*Melitaea cinxia*) revealed that populations inhabiting highly fragmented landscapes had significantly reduced genetic diversity before extinction [41]. Although this study detected selection for genotypes associated with good colonization capacity in the highly fragmented landscape before extinction, evolutionary changes in those traits did not fully compensate for the direct mortality due to habitat changes, and evolutionary rescue did not occur [41]. However, a recent review on the effects of fragmentation supports the view that evolutionary response of dispersal traits to habitat changes are likely, but are often insufficient to rescue natural populations [42]. Other forms of human-induced habitat change such as eutrophication have pronounced ecological effects that surely lead to evolutionary change through impacts on natural selection regimes, interference with sexual selection, and elevated hybridization and genetic homogenization via the breakdown of reproductive barriers [43].

### Climate change, pollution, and invasive species

Recent changes in climate [44] have profoundly affected natural ecosystems and present a new suite of selective drivers [5, 7]. For example, icing now limits access to food for several vertebrates in high arctic ecosystems [45], while in southern locations, milder springs have increased the period of vegetation growth leading to greater food abundance [46]. Merila and Hendry’s review [47] including studies on plants, insects, and vertebrates indicates that genetic adaptation to climate change has been found in some systems [48] but it is generally weaker than the contribution of plasticity to the phenotypic response to climate change [6, 21, 47]. Environmental changes often lead to apparently strong selection on demonstrably heritable traits in wild populations, but trait dynamics often do not follow evolutionary predictions [49]. This raises further questions about the extent to which human-induced changes lead to predictable evolutionary impacts. Evolutionary change may be masked by phenotypic plastic changes [50], responses may be genetically constrained by selection on correlated traits [51, 52], and the relationship between trait values and fitness may be entirely environmental in origin if selection acts on non-heritable components of trait variation [53]. Bonnet et al. [54] recently showed that despite a positive association between adult body mass and fitness in alpine snow voles (*Chionomys nivalis*), there has been microevolution towards lower body mass. In this case, there was an adaptive response to viability selection favoring juveniles that complete their development early and become relatively small adults. This selection recently intensified due to a shorter snow-free season, a consequence of changes in snowfall patterns [52]. This suggests that rapid microevolutionary response to changes in climate may go undetected if they are masked by phenotypic plasticity and non-genetic covariance with fitness. Cryptic microevolution in human-altered environments may therefore be widespread, but challenging to detect.

Genetic changes in response to pollution are also likely to be widespread based on available empirical evidence [55]. Indeed, researchers have argued that because selection pressures related to pollution involve novel chemicals, pre-existing adaptive plasticity to pollution is unlikely [15]. If this is true, one can expect that species that have persisted in a polluted environment are more likely to show local adaptation to these novel conditions. Consistent with this expectation, 56% of the studies that have documented phenotypic changes in response to pollution present evidence that those changes have a genetic basis (Fig. 3). Recent studies on fish have also revealed molecular genetic evidence for adaptation to various environmental pollutants [56, 57].

Microevolution by invasive species, and by native species in response to invasion, has also been documented [19, 58–60]. For example, it has been shown that microevolution can accelerate invasions [61] and that recently introduced invasive species impose new selective pressures on native species in the wild [62]. A classic example of human-induced co-microevolution follows the invasion of Australia by cane toads (*Rhinella marina*). The cane toad invasion is characterized by evolution of growth and dispersal rates of the invader as well as behavioral and morphological changes in the native taxa [58]. Similar to the challenges posed by pollutants, a high proportion of studies have documented a genetic basis to the changes associated with invasive species (Fig. 3), perhaps because the novel selective challenges posed by invaders make pre-existing adaptive plasticity less likely.

## The burden of proof for human-induced microevolution

Although some studies convincingly demonstrate how human-driven environmental changes can impact evolutionary dynamics in the wild, there is clearly a considerable burden of proof that needs to be met in order to unequivocally demonstrate microevolution [63]. This may be partly because there is some scepticism as to whether most forms of human activity can generate sufficiently strong selection to result in evolutionary changes that have significant effects on individual fitness and population persistence. Some authors have argued that evolutionary changes are typically insignificant relative to plasticity and can be ignored in favor of simpler models that ignore evolution [64–66]. However, it may be premature to make generalizations about the relative importance of evolutionary change based on predictions from a few poorly parameterized models [67–69] because studies that incorporate genetic data remain a minority. For example, in a recent meta-analysis evaluating the effect of urbanization on eco-evolutionary dynamics in wild species that included more than 1600 time series documenting trait changes, only 32% of those had information on the genetic basis of those traits [70] (see also Fig. 3). More importantly, while it is relatively easy to observe and document changes in demography, distribution, and extinction, this is not the case for more subtle and relatively gradual microevolutionary change, even though it may be widely taking place [70]. While it is often clear that many of the prerequisites for human-induced microevolution are present in populations under the human footprint, it can be hard to demonstrate evolutionary changes in nature because detailed information on the environmental drivers, as well as the phenotypic and genetic responses to it, are needed. Even with adequate data, it can be difficult to disentangle microevolution from phenotypic plasticity [21, 47] and trait dynamics often do not follow evolutionary predictions [49]. In the absence of compelling data, temporal phenotypic change should not be assumed to be completely evolutionary.

**Fig. 5.**
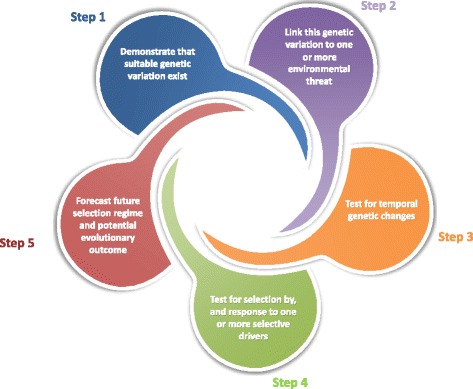
Steps required to document adaptive genetic response to environmental changes. Modified from Hansen et al. [63]

Hansen et al. [63] proposed a list of criteria that have to be met to conclude that genetic adaptation has occurred based on either molecular or quantitative trait variation (Fig. 5). Meeting some of these criteria is non-trivial. For example, demonstrating that changes in predicted breeding value over time reflect genuine microevolution requires the use of approaches that account for prediction error and genetic drift [71]. Ideally, empirical findings of human-induced microevolution based on observational study can be validated through replication or manipulations such as common garden and reciprocal transplant experiments. However, this is not always possible or logistically feasible, particularly when working on rare or endangered species and organisms with long life spans.

## Evolutionary traps, maladaptation, and detrimental microevolution

Microevolutionary responses to human-driven environmental changes are possible and, in some cases, these changes can be adaptive if they allow organisms to cope with new environmental conditions. Evolutionary changes in life-history, morphological, and behavioral traits in response to human activities may also move correlated traits away from their naturally selected optima. Given that local adaptations are widespread in nature [72], human-driven environmental changes alter the historical adaptive landscape, leading to maladaptation (i.e., a lack of fit between a population trait and its environment [73]). Thus, past and current microevolution can lead to phenotypic–environment mismatch in at least three ways. First, if plasticity is not sufficient, the initial population response to novel environmental conditions will inevitably be maladaptive because of the demographic costs of selection. For example, consider a population that is exposed to a lethal chemical in which some individuals possess a mutation that allows them to metabolize this compound. Only those individuals possessing the mutation will survive and eventually reproduce. Thus, the demographic cost of selection, manifest as the initial rate of population decline, is a consequence of the degree of mismatch between the population trait and the new environmental conditions [74]. Second, human activities may change the environment in such a way that the environmental cues used by organisms, adaptations tuned by natural selection over several generations, may no longer match resources that lead to the highest fitness [75]. Indeed, an increasing number of studies show that the fitness return of preferred novel resources is lower than historically available resources. For example, a large decline in native snakes and lizards was observed following the invasion of Australia by the cane toad because this new prey was selected by native predators although it was highly toxic [76]. Mismatches in the cue-response systems are called evolutionary traps and are a form of maladaptation [75, 77]. Alternatively, mismatch between phenotype and environment may emerge when microevolution is not fast enough to keep up with environmental changes. Temporal mismatches have been documented in several cases, including climate change [78]. Managing these mismatches is a major challenge to conservation [78]. Finally, recent studies on adaptation to environmental pollutants have documented evolutionary responses in the opposite direction of the expected outcome. For example, studies on the plankton *Daphnia ambigua* revealed that populations exposed to harmful levels of copper and cadmium evolved greater susceptibility to those chemicals [79]. While these results are puzzling and the mechanism behind it unknown, they underline the challenges that researchers will face in trying to predict evolutionary responses to human threats.

## Key tool for the future: genomics

Genomics provides a critically important set of tools for detecting, monitoring, and predicting the evolutionary consequences of human activity on wild populations at a molecular level [63, 80, 81]. Genomic data enable avenues for research in wild populations. For example, genomics obviates the need to develop pedigrees for estimating quantitative genetic parameters and predicting the response to selection, as relatedness matrices can be directly estimated from genomic data [82]. Genomics also provides methods for assaying epigenetic changes at a molecular level, and epigenetic mechanisms likely play key roles in phenotypic plasticity through their effects on gene expression [83]. The recent development of low-cost, high-throughput DNA sequencing methods has perhaps made it more feasible to collect high quality genomic data on large numbers of individuals than it is to collect high quality phenotypic data for some wild species [84], although it is clearly no substitute for the collection of phenotypic data. Detection of changes in neutral genetic diversity that may occur in response to human-induced impacts on population demography or connectivity is commonplace [25, 85] and has even been recently shown using genome-wide markers [86] and whole genome sequence data [87]. The human footprint on intraspecific biodiversity may be less well appreciated than it is on extinction [88]. However, convincing demonstration of local adaptation and microevolutionary responses using genomic data is much more challenging [89].

This demonstration requires the identification of loci significantly associated with environmental (e.g., outliers or allele–environment association) and/or trait variation (QTL mapping or genome-wide association) linked to variability in individual fitness or coupled with observed changes in allele frequency over time. This can be achieved through typing historical samples or using samples collected across replicated spatial–environmental gradients. Here it is critical that changes over time or space have adequately controlled for confounding effects of drift and spatial autocorrelation [90]. Ideally, loci showing evidence for microevolution can also have their effects validated in independent analyses or through functional characterization under appropriate conditions. Functional analysis may also provide insights into the mechanisms underlying a micro-evolutionary response. For example, the mutation responsible for the iconic example of industrial melanism in the peppered moth (*Biston betularia*) has recently been mapped to the insertion of a transposable element into the first intron of a gene that plays an important role in cell-cycle regulation during early wing disc development [91].

Functional analyses are, however, not always feasible or even possible in some systems and, with respect to validation, it is also possible that micro-evolutionary changes at the genomic level may be less repeatable and therefore predictable than they are at the phenotypic level due to the complexity of genomic architectures [92, 93]. Furthermore, the genomic architecture of many quantitative traits that are likely to be affected by human activity is likely to be highly polygenic [80, 94] (but see [95, 96]), making the detection of temporal changes and the validation of microevolution at the genomic level extremely challenging. There is clearly a need to develop and utilize polygenic models of adaptation, phenotypic prediction, and forecasting of future trends that consider large numbers of small effect loci rather than standard models of single genes of major effect [80, 97]. These approaches need to be incorporated in eco-evolutionary dynamic models to better advance our understanding of how human-mediated impacts are likely to impact biodiversity [98, 99].

## Key challenge: forecasting eco-evolutionary dynamics

Detecting microevolution is only a starting point—we need tools and approaches for monitoring the rate of change [63] and predicting the impact of human-induced microevolution [80]. So far, most eco-evolutionary analyses have retrospectively explained what has already happened. Some branches of science can successfully predict the future. Astronomy, for example, can precisely predict when a comet will pass close to the Earth. Evolutionary ecology, however, has yet to build a predictive framework [92]. Given that it is already difficult to document and predict contemporary evolution, as heritable traits under apparently strong directional selection often fail to show the expected evolutionary response [49], it will be very challenging to achieve this. Improved evolutionary predictability, particularly as it relates to human stressors, is an area of major concern in urgent need of resolution [54]. However, we can try to apply models that will tell us under what circumstances inherited or plastic trait changes are likely to occur, and develop more robust models integrating the genetic basis of traits that can help forecast the consequences of trait changes on population processes [80]. Although ecological models, such as integral projection models which can predict how changes in phenotype affect population growth over the short and long term, offer powerful opportunities to link genotype and phenotype to individual and population performance, the proper integration of quantitative genetics is still needed [67, 68]. An important step forward in predicting adaptation to human-induced challenges will be to fully harness genomic data and ecological information in an integrative framework such as the evolutionary response architecture proposed by Bay et al. [80]. This concept proposes that evolutionary prediction should be based on the integration of knowledge of genetic architecture, spatial heterogeneity, phenotypic plasticity, and population dynamics.

Given increasing evidence of trait changes driven by human activity, it is critical to document the consequences of trait changes (plastic or genetic) on population dynamics, community, and ecosystem function in wild species, especially those under human threats. For example, documenting eco-evolutionary dynamics, such as whether evolutionary rescue occurs frequently in nature, is central to understanding how species may cope with large-scale human-driven environmental changes characteristic of the Anthropocene. A recent review by Mimura et al. underlined the importance of understanding and monitoring the effect of human-driven changes on intraspecific variation—a major component of biodiversity [88]. However, we see that documenting microevolution in nature is not a simple task, and few studies have met the burden of proof required. Thus, the importance of microevolution to population persistence is still unknown and is likely to fluctuate [100].

A potentially good starting point is to investigate the consequence of phenotypic changes at higher levels of biological organization and, in parallel, try to evaluate whether those trait changes are driven by genetic changes or plasticity. Indeed, several researchers have argued that trait changes caused by human activity may be shaping ecological dynamics on a global scale [14]. Since traits change in response to human activities approximately twice as fast as they respond to other drivers [11], the first step might be to use trait changes in forecasting models to tackle two questions: 1) under what circumstances are trait changes more likely to happen and 2) what are the ecological consequences of those changes? Given that it is the overall phenotypic change that is likely to feed back on ecological dynamics [14, 101], generalization of the circumstances favoring trait changes and their consequences will be very useful. Having said that, we ought not to focus solely on phenotypic variation because plastic and genetic changes occur on different time scales and the latter may be more difficult to reverse [34]. This interplay will impact our predictions for population persistence on the short and medium time-scale. Evolutionary demographic approaches that integrate both traits and demographic information could be useful tools to tackle these questions and make predictions on population parameters in the presence or absence of evolution [102], as long as genetic transmission is correctly integrated [68].

Another useful avenue may be to use intraspecific genetic and phenotypic changes to monitor human impacts on wild populations [88]. Indeed, trait changes could be integrated as early warning signs of population collapse [103]. Clements and Ozgul [103] showed that including phenotypic information on body size in composite early warning indices can more accurately predict critical transitions in population dynamics than using abundance time-series alone. This framework could easily be expanded to include intraspecific data on fitness-related genetic variation. So far, however, composite early warning signals have only been applied in controlled environments and it is yet to be determined whether they can be used to detect bifurcations in population dynamics (major increase or collapse) in the wild.

## Outlook: the need for a predictive framework

It is clear that human activity has profound effects on the eco-evolutionary dynamics of wild populations via novel and strong selection pressures, and populations affected by these pressures show evidence of rapid phenotypic change. While the prerequisites for evolutionary response are often present in human-altered environments, the extent to which these changes generally represent microevolution versus plasticity is uncertain. A recent review found that global levels of threat are increasing by 1 to 2% per decade for populations of birds and mammals where systematic monitoring data are available [104]. Based on current knowledge of threats to biodiversity, they also suggest that the rate of extinction could soon rise to at least five times higher than it has been in the recent past. Thus, there is an urgent need for more empirical studies investigating multiple drivers to gain a better understanding of contemporary microevolution in response to multiple human-driven environmental changes so that eco-evolutionary dynamic models can be better parameterized using phenotypic and genetic data. It is also critical that we move towards a predictive framework that integrates the fitness consequences of plasticity and contemporary microevolution to forecast the fate of natural populations affected by human activity and other stresses. Such research should be of high priority in the Anthropocene era in which human activity is a key driver of intraspecific variation with widespread implications for natural resource management, ecosystem services, and biodiversity conservation.
